# Supplementary material for: Potential for Drug Repositioning of Midazolam for Dentin Regeneration
Source: Int J Mol Sci. 2019 Feb 4;20(3):670. doi: 10.3390/ijms20030670 (PMC6387224; doi:10.3390/ijms20030670)
Supplement: Supplementary file 1 [file ijms-20-00670-s001.pdf]

**Supplementary Materials**

# Potential for Drug Repositioning of Midazolam for Dentin Regeneration

**Takeo Karakida [1], Kazuo Onuma [2], Mari M. Saito [1], Ryuji Yamamoto [1], Toshie Chiba [3], Risako Chiba [1], Yukihiko Hidaka [4], Keiko Fujii-Abe [4], Hiroshi Kawahara [4] and Yasuo Yamakoshi [1,*]**

[1] Department of Biochemistry and Molecular Biology, School of Dental Medicine, Tsurumi University, 2-1-3 Tsurumi, Tsurumi-ku, Yokohama 230-8501, Japan; karakida-t@tsurumi-u.ac.jp (T.K.), saito-mari@tsurumi-u.ac.jp (M.S.), yamamoto-rj@tsurumi-u.ac.jp (R.Y.), chiba-r@tsurumi-u.ac.jp (R.C.)

[2] National Institute of Advanced Industrial Science & Technology, Central 6 1-1-1 Higashi, Tsukuba, Ibaraki 305-8566, Japan; k.onuma@aist.go.jp (K.O.)

[3] Research Center of Electron Microscopy, School of Dental Medicine, Tsurumi University, 2-1-3 Tsurumi, Tsurumi-ku, Yokohama 230-8501, Japan; chiba-t@tsurumi-u.ac.jp (T.C.)

[4] Department of Dental Anesthesiology, School of Dental Medicine, Tsurumi University, 2-1-3 Tsurumi, Tsurumi-ku, Yokohama 230-8501, Japan; 2911002@stu.tsurumi-u.ac.jp (Y.H.), fujii-keiko@tsurumi-u.ac.jp (K.A.), kawahara-h@tsurumi-u.ac.jp (H.K.)

* Correspondence: yamakoshi-y@tsurumi-u.ac.jp; Tel: +81-45-580-8479; Fax: +81-45-573-9599 (Y.Y.)

## *1. Establishment of cell line from porcine dental pulp cells*

Dental pulp cell lines have been generated and characterized from various mammalian species, but a few studies have been conducted to characterize dental pulp cells-derived spheroids [1-4]. As a first step, we established porcine dental pulp-derived cell lines.

Tooth germs of permanent incisors were surgically extracted from the mandibles of deceased 5-month-old pigs (n = 10) from the Meat Market of Metropolitan Central Wholesale Market (Shinagawa, Tokyo, Japan). Pulp tissue pulled out from tooth germs was briefly rinsed in ice-cold sterile PBS to remove blood cells, minced with a surgical blade and digested in a solution of 0.1% collagenase and 0.2% dispase II (Wako Pure Chemical Industries, Osaka, Japan) for 1 hr at 37°C with gentle shaking. The released cells were passed through a 70-μm cell strainer (BD Falcon, Bedford, MA, USA) and washed three times with PBS by centrifugation. The cells were then cultured in alpha Minimum Essential Medium ($\alpha$MEM) (Gibco/Life Technologies, Carlsbad, CA, USA) containing 10% fetal bovine serum (FBS) and antibiotics (50 U $mL^{-1}$ of Penicillin, 50 μg $mL^{-1}$ of Streptomycin, Gibco) at 37°C in a humidified 5% $CO_2$ atmosphere.

Pulp cells isolated from porcine tooth germs were plated at subconfluent cell densities and transfected with the pSV3-neo plasmid (ATCC 37150) using Lipofectamine 2000 (Invitrogen/Life Technologies, Carlsbad, CA, USA) in accordance with the manufacturer's instructions. The pSV3-neo plasmid expresses the SV40 large T-antigen for immortalization and neomycin phosphotransferase for selection. Two days after transfection, the cells were re-plated at low density and selected with 0.35 mg $mL^{-1}$ of geneticin (G418) (ATCC, Manassas, VA, USA). The cells were cultured in media containing G418 until colonies were visible. Individual colonies were isolated with cloning cylinders and maintained in the standard medium at 37°C in a humidified 5% $CO_2$ atmosphere.

Following the transfection with pSV3-neo plasmid, we incubated over 20 G418-resistant colonies. However, as most of the cell growth was stopped, we selected 8 clones (PPU-1, -3, -7, -10, -12, -16, -17 and -18). The immortalized pulp cells' morphology was fibroblast-like (Figure S1a).

Since the alkaline phosphatase (ALP) has been believed as the initial marker for the

differentiation of mesenchymal cells into hard tissue-forming cells such as osteoblasts or odontoblasts [5,6], we determined the inherent ALP activity of the 8 immortalized pulp cell lines. Both PPU-3 and PPU-7 cell lines possessed the high inherent ALP activity, whereas the PPU-16 cell line had a trace of ALP activity (Figure S1b). We further investigated the effects of BMP2 and TGF-β1 on ALP activity of 8 immortalized pulp cells along with the determination of inherent ALP activity. The addition of BMP2 enhanced an increasing ratio of ALP activity in PPU-10 (~1.5-fold) and PPU-16 (~2.2-fold) cell lines, when the activity of control (i.e. 8 immortalized pulp cells were cultured without any cytokines) was 1 (Figure S2a). When we cultured those two cell lines with BMP2 and LDN-193189, which is a selective BMP signaling inhibitor, the ALP activity level was decreased to below baseline. ALP activity of other cell lines (PPU-1, -3, -7, -12, -17 and -18) in the addition of BMP2 was almost baseline levels. Although PPU-16 possessed the lowest ALP activity of the 8 clones, it showed the highest increase in ALP activity in response to BMP2. In contrast, the addition of TGF-β1 was decreased ALP activity in each pulp cells below baseline (Figure S2b). When we cultured each pulp cells with TGF-β1 and SB431542, which is a specific and selective inhibitor of TGF-β type I activing receptor-like kinase (ALK) receptors, the ALP activity was restored to near baseline levels. Based on these experimental results, we selected two cell lines: PPU-7, possessing the high inherent ALP activity, and PPU-16, showing the high reactivity for BMP2.

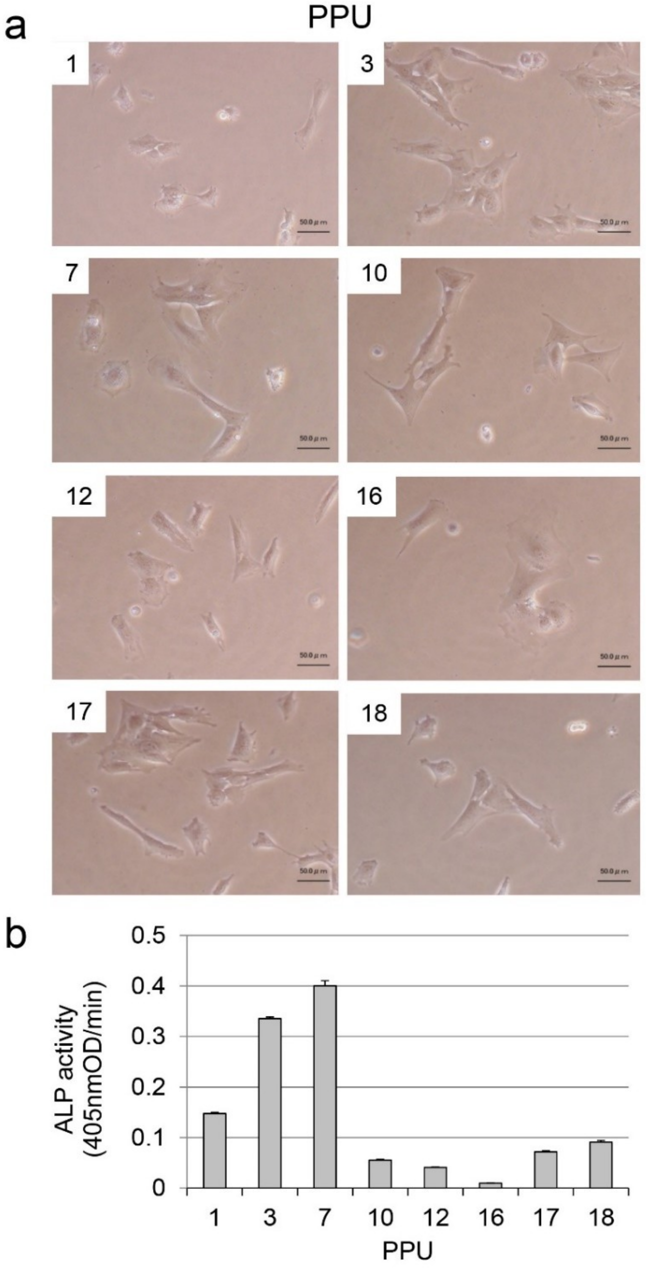


**Figure S1.** Morphology and inherent alkaline phosphatase (ALP) activity of porcine dental pulp-derived cell lines. **(a)** Eight dental pulp-derived cell lines (PPU-1, -3, -7, -10, -12, -16, -17 and -18) are shown. Bars = 50 µm. **(b)** Inherent ALP activity of the respective cell lines. Data bars are means ± standard error (s.e.m.) of 6 culture wells.

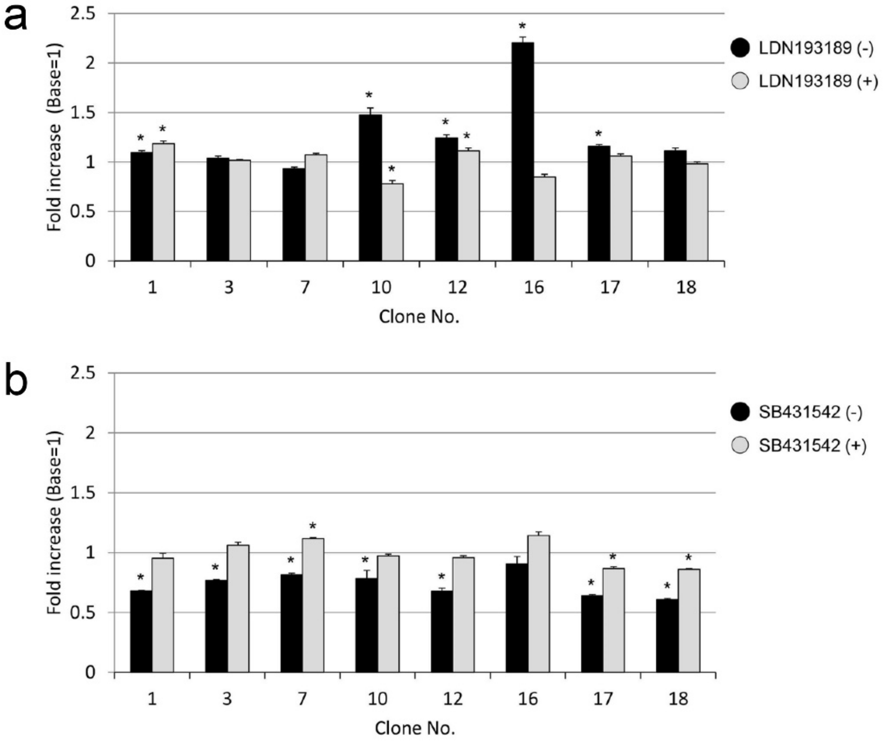


**Figure S2.** Effect of BMP2 and TGF-β1 on inherent ALP activity of porcine dental pulp-derived cell lines. Effect of **(a)** BMP2 with or without LDN-193189, and **(b)** TGF-β1 with or without SB431542. Values of inherent ALP activity are indicated as the fold increase, when the activity of the control was 1. Data are means ± standard error (s.e.m.) of 6 culture wells (*$p<0.05$).

*2. Cell Proliferation Rate*

We investigated the effects of BMP2 and TGF-β1 on cell proliferation in the PPU-7 and PPU-16 cell lines and expressed cell density using a semi-logarithmic scale to judge the logarithmic growth phase by linearity (Figure S3a). TGF-β1 treatment inhibited the cell proliferation in both PPU-7 and PPU-16 cell lines, whereas BMP2 treatment inhibited the cell proliferation in PPU-7 cell line only. No significant differences were observed between the control and BMP2 treatment in the PPU-16 cell line. Compared with the control, the cell population doubling time of PPU-7 increased significantly with BMP2 or TGF-β1 treatment (Figure S3b). TGF-β1 treatment significantly increased the doubling time of the PPU-16 cell line, but BMP2 treatment did not (Figure S3b).

*3. Gene Expression in PPU-7 and PPU-16 Cell Lines*

Gene expression of a panel of odontoblastic markers in both PPU-7 and PPU-16 cell lines at 1, 3 and 7 days following BMP2 or TGF-β1 treatments was analyzed by qPCR (Figure S3c). We amplified two products from the full-length dentin sialophosphoprotein (*Dspp*) transcript: a segment containing the dentin glycoprotein (DGP) + dentin phosphoprotein (DPP) coding region (*DSPPv1*), and a smaller segment specific for the dentin sialoprotein (DSP)-only transcript (*DSPPv2*). In addition to those dentin markers, we checked the expression of *ALP* as the initial marker for the differentiation of dental pulp cells. Expression levels of *DSPPv1, DSPPv2,* and *ALP* in PPU-7 cell line were higher than those in PPU-16 cell line throughout the culture period. Comparing the effects of BMP2 and TGF-β1, both BMP2 and TGF-β1 treatments up-regulated

both DSPPv1 and DSPPv2 expression levels in PPU-7 cell line at 7 days. Both BMP2 and TGF-β1 treatments also gradually up-regulated ALP expression level in the PPU-7 cell line, but TGF-β1 treatment did not affect the PPU-16 cell line.

Thus, based upon above results, we selected PPU-7 cell line and used to find the potential for drug repositioning of MDZ toward dentin regeneration at genetic, protein and crystal engineering levels.

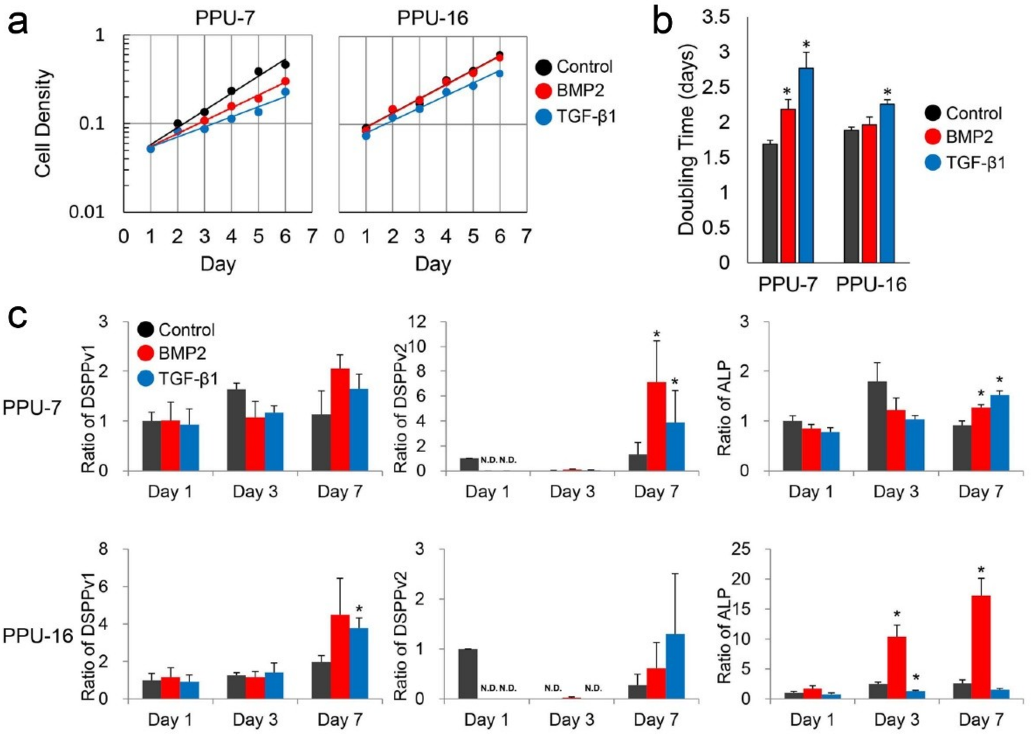


**Figure S3.** Effect of BMP2 and TGF-β1 on proliferation and gene expression of PPU-7 and PPU-16 cell lines. **(a)** Cell density plotted on a semi-logarithmic scale was used to judge the logarithmic growth phase by linearity, which was determined in turn every day for 6 days. **(b)** Cell population doubling level against days in culture following transfection. Data are means of ± standard error (s.e.m.) of 6 culture wells. BMP2 and TGF-β1 in PPU-7 or BMP2 in PPU-16 significantly required time (*$p<0.05$). **(c)** The mRNA expression by qPCR analysis of DSPP-variant 1 (DSPPv1, left), DSPP-variant 2 (DSPPv2, middle), and ALP (right). Each ratio was normalized to glyceraldehyde-3-phosphate dehydrogenase (GAPDH) as a reference gene, and the relative quantification data of DSPPv1, DSPPv2, and ALP in PPU-7 or PPU-16 were generated on the basis of a mathematical model for relative quantification in a qPCR system (n = 6 PPU-7 or PPU-16) All expression levels are indicated as the increasing or decreasing ratio, when the mRNA level of control (Day 1) was 1. Data are means ± standard error (s.e.m.) (* $p<0.05$). N.D.: not determined.

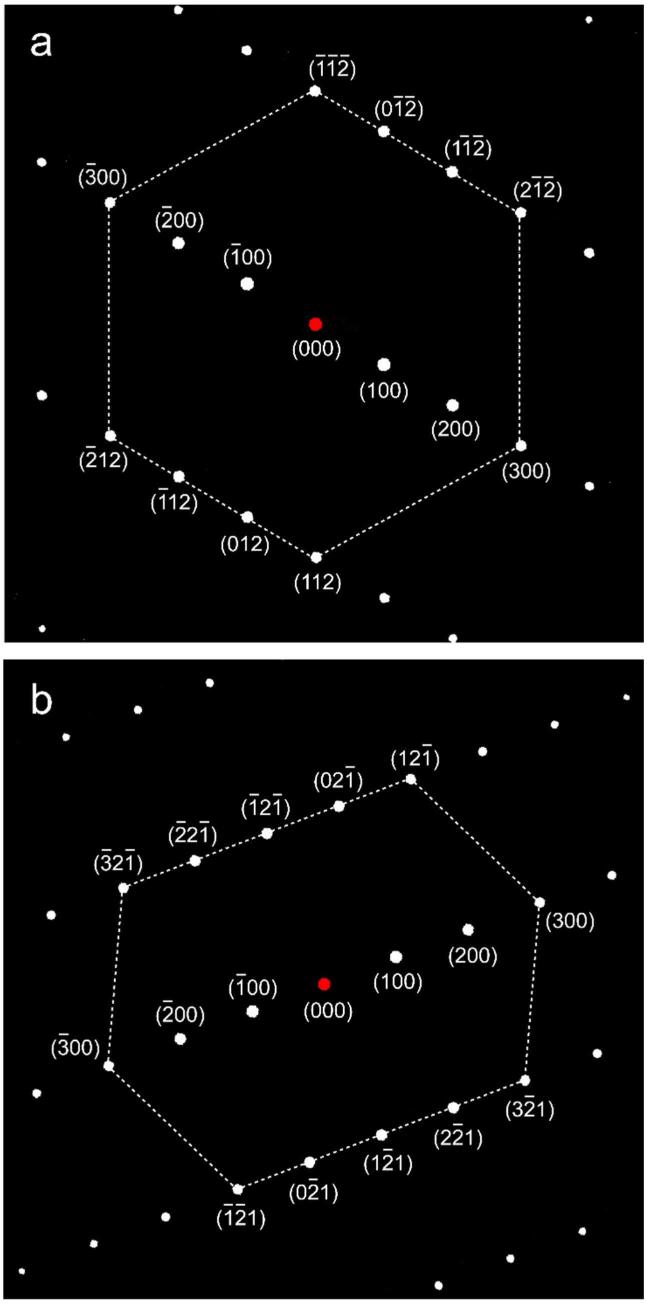


**Figure S4.** Simulated SAED patterns of HAP crystals viewed from **(a)** [02$\bar{1}$] and **(b)** [012] zone axes. Two dotted hexagons correspond respectively to FFT images in main Figure 8c and Figure 9c.

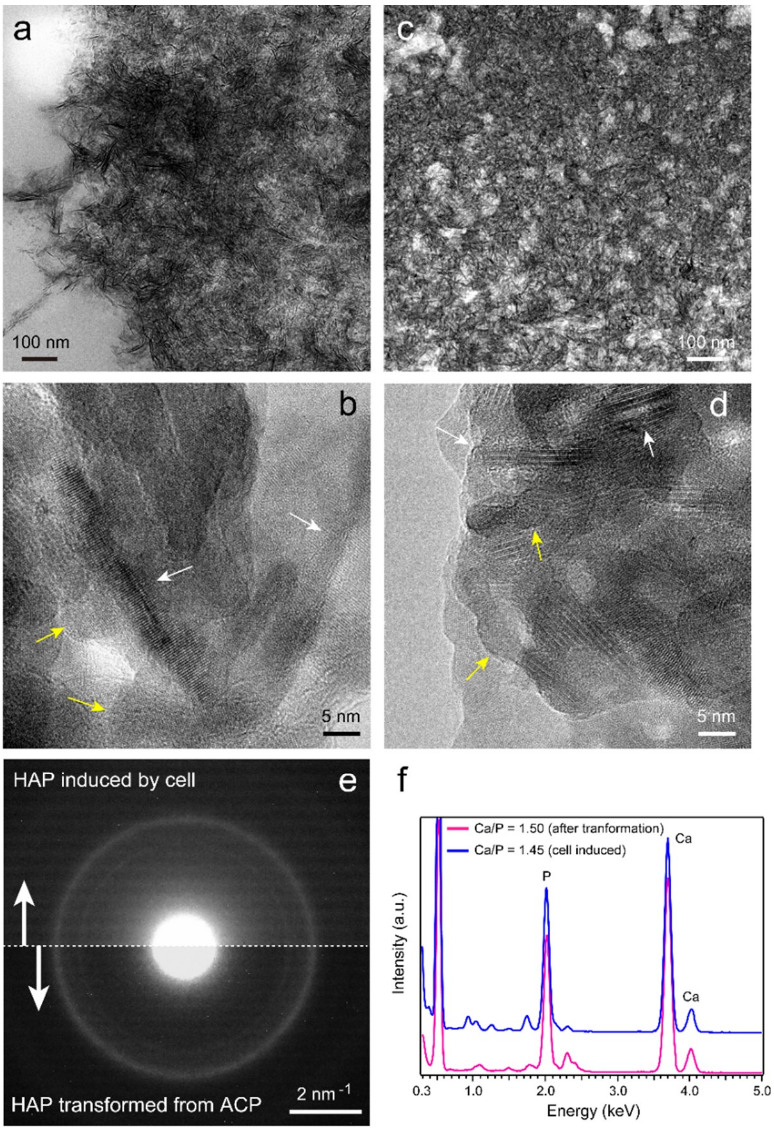


**Figure S5. (a)** TEM image of cell-induced precipitates. Fibrous morphology was observed. **(b)** High-magnification image corresponding to **(a)**. Precipitates consisted of nanorods (white arrows) and nanoparticles (yellow arrows), which were randomly oriented. **(c)** Macroscopic TEM image of ACP substrate after transformation. Sample was prepared by focused ion beam milling. Fibrous morphology similar to that in **(a)**. **(d)** High-magnification image corresponding to **(c)**. As for **(b)**, transformed substrate consisted of randomly oriented nanorods (white arrows) and nanoparticles (yellow arrows). **(e)** Superimposed SAED patterns of cell-induced precipitates (upper region) and ACP-transformed substrate (lower region). Patterns are consistent. **(f)** STEM-EDS spectra for cell-induced precipitates (blue curve) and ACP-transformed substrate (magenta curve).

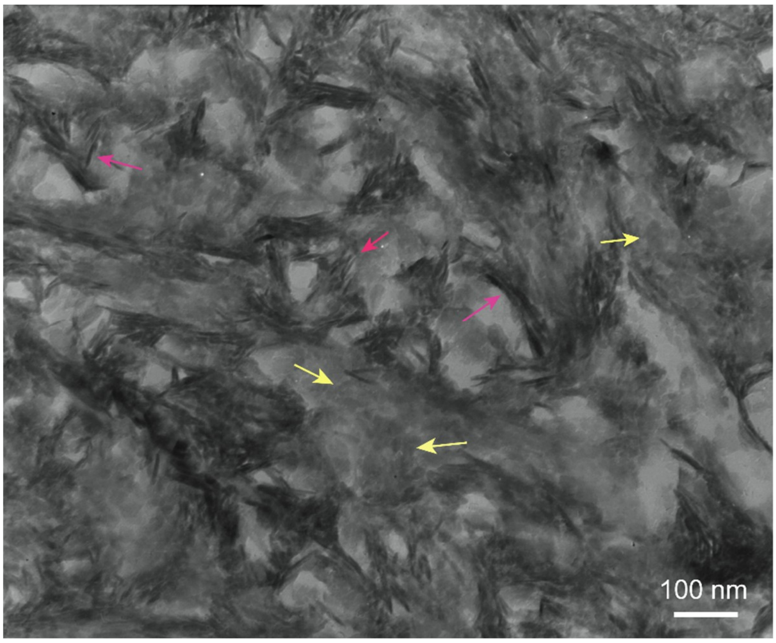


**Figure S6.** TEM image of human molar dentin. Magenta and yellow arrows indicate HAP nanorods and nanoparticles, respectively, which are similar to those observed in Figure S5a and S5c.

**Table S1.** Selected primers, size of amplified product for qPCR analysis shown in main Figure 2.

| Gene | | Sequence (5'->3') | Size (bp) | qPCR Protocol (45 cycles) |
|---|---|---|---|---|
| *Dspp-v1* | F | CCCAGAAACCCAATCAGAGA | 300 | Denaturation 95 °C 10 sec<br>Annealing 60 °C 10 sec<br>Extension 72 °C 15 sec |
| | R | TATGTGTTTTGCTGGGTCCA | | |
| *Dspp-v2* | F | CCCAGAAACCCAATCAGAGA | 149 | |
| | R | GGGAAGGAAGGGGAGAATTT | | |
| *Mmp2* | F | CCGACGTGGCCAATTACAAC | 96 | |
| | R | GGTCCAGATCAGGCGTGTAG | | |
| *Runx2* | F | CAACTTCCTGTGCTCTGTGC | 119 | |
| | R | CCGCCATGACAGTAACCACA | | |
| *OC* | F | CCAGGCAGATGCAAAGCCTA | 96 | |
| | R | CGCCTGAGTCTCTTCACCAC | | |
| *Col II* | F | CCAGATTGAGAGCATCCGCA | 142 | |
| | R | CATGGCGTCCAAAGTGCATC | | |
| *ACAN* | F | ACAACGCTCAGGACTACCAG | 134 | |
| | R | TCCAGTGGCGAAGAAGTTGT | | |
| *Gapdh* | F | CCATCACCATCTTCCAGGAG | 346 | |
| | R | ACAGTCTTCTGGGTGGCAGT | | |

F: forward, and R: reverse

**Table S2**. Ionic concentration of culture fluid and supersaturation for each calcium phosphate phase.

| ion | concentration (mM) | calcium phosphate phase | supersaturation |
|---|---|---|---|
| Ca | 1.967 | DCPD | 1.58 |
| Na | 163.2 | | |
| K | 5.824 | OCP | 6.42 |
| Mg | 75.73 | | |
| P | 11.20 | β-TCP | 7.46 |
| Cl | 123.2 | | |
| $SO_4$ | 0.279 | HAP | 25.0 |
| $CO_3$ | 23.57 | | |

**Table S3.** Comparison of interplanar distance and intersection angle between measured and theoretical values for HAP nanorod and nanoparticle.

Nanorod

| plane | *d* measured (nm) | theoretical (nm) | angle with [100] measured (degree) | theoretical (degree) |
|---|---|---|---|---|
| (100) | 0.814 | 0.816 | | |
| (112) | 0.277 | 0.278 | 59.02 | 59.27 |
| ($\bar{2}$12) | 0.276 | 0.278 | 120.3 | 120.7 |

Nanoparticle

| plane | *d* measured (nm) | theoretical (nm) | angle with [100] measured (degree) | theoretical (degree) |
|---|---|---|---|---|
| (100) | 0.809 | 0.816 | | |
| (1$\bar{2}$1) | 0.385 | 0.388 | 90.66 | 90.00 |
| (3$\bar{2}$1) | 0.281 | 0.281 | 46.96 | 46.38 |

**Table S4.** Elements and content in cell-induced precipitate measured by STEM-EDS.

| element | content (atomic %) | error (atomic %) |
|---|---|---|
| Ca | 23.56 | 2.17 |
| P | 16.30 | 1.53 |
| O | 56.17 | 5.18 |
| Na | 0.83 | 0.16 |
| Mg | 0.55 | 0.13 |
| Al | 0.23 | 0.09 |
| Si | 1.65 | 0.10 |
| S | 0.58 | 0.12 |
| Zn | 0.12 | 0.04 |

## References


1. Xiao, L.; Kumazawa, Y.; Okamura, H. Cell death, cavitation and spontaneous multi-differentiation of dental pulp stem cells-derived spheroids *in vitro*: A journey to survival and organogenesis. *Biol. Cell* **2014**, *106*, 405-419.
2. Yamamoto, M.; Kawashima, N.; Takashino, N.; Koizumi, Y.; Takimoto, K.; Suzuki, N.; Saito, M.; Suda, H. Three-dimensional spheroid culture promotes odonto/osteoblastic differentiation of dental pulp cells. *Arch. Oral Biol.* **2014**, *59*, 310-317.
3. Xiao, L.; Tsutsui, T. Characterization of human dental pulp cells-derived spheroids in serum-free medium: Stem cells in the core. *J. Cell. Biochem.* **2013**, *114*, 2624-2636.
4. Karbanova, J.; Soukup, T.; Suchanek, J.; Pytlik, R.; Corbeil, D.; Mokry, J. Characterization of dental pulp stem cells from impacted third molars cultured in low serum-containing medium. *Cells Tissues Organs* **2011**, *193*, 344-365.
5. Sloan, A.J.; Rutherford, R.B.; Smith, A.J. Stimulation of the rat dentine-pulp complex by bone morphogenetic protein-7 in vitro. *Arch. Oral Biol.* **2000**, *45*, 173-177.
6. Chen, S.; Gu, T.T.; Sreenath, T.; Kulkarni, A.B.; Karsenty, G.; MacDougall, M. Spatial expression of Cbfa1/Runx2 isoforms in teeth and characterization of binding sites in the DSPP gene. *Connect. Tissue Res.* **2002**, *43*, 338-344.
